# Disabling hearing loss prevalence in Juiz de Fora, Brazil

**DOI:** 10.1590/S1808-86942012000400011

**Published:** 2015-10-20

**Authors:** Débora Modelli Baraky, Ricardo Ferreira Bento, Nádia Rezende Barbosa Raposo, Sandra Helena Cerrato Tibiriçá, Luiz Cláudio Ribeiro, Marcelo M.V.B. Barone, Nátalia Baraky Vasconcelos

**Affiliations:** PhD (Professor at the Medical Residency program in Otorhinolaryngology - Medical School of the Federal University of Juiz de Fora - UFJF); Full Professor of Otorhinolaryngology - Medical School of the University of São Paulo - FMUSP; Department of Ophthalmology and Otorhinolaryngology - University of São Paulo - SP, Brazil); Post-Doctorate (Adjunct Professor IV of Toxicology - Pharmacy School - UFJF); PhD (Professor at the Department of General Practice - Medical School of the Federal University of Juiz de Fora - MG, Brazil); PhD (Professor of Statistics - UFJF); MD. Otorhinolaryngologist - Federal University of Juiz de Fora - UFJF - MG, Brazil); Medical Student - School of Medical and Health Sciences of Juiz de Fora)

**Keywords:** deafness, hearing loss, public health

## Abstract

Data on the prevalence of disabling hearing loss (DHL) in Brazil is scarce, which impacts healthcare professionals' knowledge on the extent of the problem.

**Objectives**: This study aimed at estimating DHL prevalence in the municipality of Juiz de Fora, Minas Gerais, to identify individual-related variables and find risk areas.

**Materials and Methods**: This was a descriptive sectional population study held from January to October of 2009. We randomly selected 349 households with 1,050 individuals who with ages ranging between 4 days and 95 years. The data collection instruments were: WHO structured questionnaire, ENT examination and laboratory tests. Chi-square and Poisson regression models were used for analyses.

**Results**: DHL prevalence was estimated at 5.2% (95% CI = 3.1 to 7.3) which was classified as moderate in 3.9% (95% CI = 0.001 to 0.134), severe in 0.9% (95% CI = 0.001 to 0.107) and profound in 0.4% (95% CI = 0.001 to 0.095). We found correlation between DHL and tinnitus; age over 60 years and low educational level.

**Conclusions**: Our data obtained pointed to the need to create hearing health programs targeted to specific risk groups, promoting quality of life for hearing impaired patients.

## INTRODUCTION

Verbal communication skills is a distinctive trace of the human race with undisputed importance at any age range^1^. Sensorial auditory deprivation impairs the quality of life of the individuals and their families in the biological, psychological, social and environmental aspects. Disabling hearing loss (DHL) affects 278 million people all over the world, 2/3 of them in developing countries^2^ - especially during childhood. Hearing loss (HL) can cause development disorders with delays in speech, language and emotional development as well as in emotional, educational and social maturing^3^.

DHL in children (younger than 15 years of age) was defined as the permanent increase of the auditory threshold in the best ear in more than 30 dB HL and in more than 40 dB HL in adults, measured using pure tones in the frequencies of 0.5; 1; 2 and 4 KHz^4^.

Hearing loss is the most prevalent of the disorders that can be diagnosed upon birth. In one study carried out in Philadelphia - USA. congenital hearing loss was twice more prevalent (220 in 100.000 births) than all the most common metabolic disorders^5^. Since the last decade, screening and the early diagnosis of HL in newborns has been broadly used; nonetheless, in countries such as South Africa, 90% of the population does not have access to neonatal auditory screening^6^. In Brazil, Universal Neonatal Auditory Screening (UNAS) is provided by law and is carried out by the Public Health Care System - SUS^7^.

The demographic transition which has been happening in Brazil in the past decades has as one of its consequences an increase in the number of elderly persons in the population^8^. Associated to it, we also notice that individuals are living longer. These facts contribute to an increase in the incidence of HL among the elderly, in a growing prevalence along time. Such population aspects point to the importance of diagnosing HL in the elderly. given that it impacts conversation and predisposes them to depression^9^. The increase in the incidence and prevalence of HL has major economic consequences. In the USA, the costs of physiological hearing loss (presbycusis) in the age range above 65 years is close to US$ 8.2 billions per year^10^.

Contrary to the universal trend involving studies on the hearing health of babies. the efforts to track and prevent HL in the adult and elderly populations are insufficient or simply non-existent, especially concerning risk factors and comorbidities^11^. Studies have suggested that smokers and diabetics have considerable risks to develop HL and could benefit from its early diagnosis and intervention^12^. It is worth stressing that estimates or data from other countries do not fit Brazilian needs and realities.

The results obtained with this study could help healthcare managers from Juiz de Fora - MG, Brazil, subsidize strategic actions in planning and implementing hearing health programs and allocating public health resources for this end.

The data can also be utilized by the Department of Health and the World Health Organization (WHO) to plot the global HL prevalence.

Considering the epidemiological pertinence, the study aims at estimating the prevalence of disabling hearing loss (DHL) in the city of Juiz de Fora, its relationship with the different variables (gender, age, family income, race, education, and others), as well as the distribution of individuals with DHL in the seven census regions of the city.

## MATERIALS AND METHODS

### Series and experiment outline

DHL prevalence was estimated by means of a sectional population study, encompassing the age range between 4 days and 95 year, carried out between January and October of 2009, in the city of Juiz de Fora.

In order to make sure we included homes from all the urban areas of the city, we randomly chose two sectors for each one of the seven administrative regions of the city: central, east, northeast, north, west, southeast and south). The sample size (1.050 individuals) was calculated considering a 2% error margin, prevalence was estimated to be 5% and the confidence interval was 95%. We ran a random sampling procedure within each one of the sectors, collecting data from one of every five homes. We included everyone with permanent residence among the 349 homes randomly chosen who accepted to participate in the study, after they signed an informed consent form. We took off the study those people who did not live in the house visited, collective homes, commercial establishments and people with more than 95 years of age.

When the researcher did not find a qualified person at home for the interview, a new visit was scheduled - we would return three times at different hours when needed. In case of a frustrated approach on the third time, we would go to the neighboring home immediately opposite the one we had been to, then immediately behind, and so on, until we found a qualified person to be interviewed.

The collecting instruments were based on the translation of the “WHO - Ear and Hearing Disorders Survey Protocol for a Population-Based Survey of Prevalence and Causes of Deafness and Hearing Impairment and other Ear Diseases” questionnaire (WHO. 1999), an ear exam and complementary tests - including tonal audiometry or transient otoacoustic emissions (TOAEs). Data collection was carried out by two teams; each one made up of one otorhinolaryngologist, one speech and hearing therapist and three medical students. The mean time spent in data collection in a family of four was 20 minutes.

The validated questionnaire, according to the model proposed by the WHO, was slightly changed when we included some questions about comorbidities. The researchers were trained using the questionnaire handbook - also validated by the WHO, which aimed at achieving reproducibility in data collection.

The main forecasting variables were: age, gender, race, family income, education, high blood pressure, diabetes mellitus, tinnitus, smoking.

The test carried out by the otorhinolaryngologist was guided towards inspecting the ear pinna and otoscopy (otoscope model K 100® 3.5V. Welch Allyn. USA), which was carried out before the audiometric tests. The answers were written down on the clinical assessment sheet of the questionnaire. We used disposable specula. The patient had his/her characteristics maintained before the audiometry and, if needed, the intervention was carried out after data collection, such as ear wax removal in 67 patients or when they were referred for surgical procedures. As far as the otoscopy is concerned, we found the following among the individuals assessed (n = 1.050) bilateral otomycosis 2 (0.2%); unilateral foreign body 2 (0.2%); bilateral ear wax 59 (5.6%); unilateral ear wax 8 (0.7%); bilateral inflammation 3 (0.3%); bilateral eczema 2 (0.2%) in the external ear canal and bilateral edema or hyperemia 8 (0.7%); unilateral tympanic membrane hyperemia (0.1%); bilateral stiffness or retraction 7 (0.6%); bilateral perforation 3 (0.3%); tympanosclerosis 5 (0.5%); bilateral atelectasis 1 (0.1%); unilateral atelectasis 1 (0.1%).

The hearing therapist (audiologist) decided for using the TOAEs (OtoRead Portable OAE - Screener. Interacoustics®. Assens. Denmark) and a large bell agogô to screen children below 4 years of age concerning their eyelid reflex. Threshold tonal audiometry was carried out using the calibrated Diagnostic Audiometer AD 226. Interacoustics®. Assens. Denmark) in those children below 4 years of age and in the adults. Air conduction assessment was carried out in the frequencies of 1,000; 2,000 and 4,000 Hz. The test was carried out in the most silent room in the house, chosen by measuring room noise by means of a decibelimeter (4189, Bruel & Kjaer). We used the WHO's DHL classification.

The study was approved by the National Committee of Ethics in Research (# 0214/08).

### Statistical analysis

The numerous variables studied were described by means of data exploratory and descriptive techniques. The statistical significance of association between the qualitative variables studied with the auditory characteristic of the subject (having or not having disabling hearing loss) was determined by the chi-squared test (ξ^2^). The quantitative variable was described by the Student t test. The variables which had association with the auditory characteristic (*p* < 0.1) were included in the Poisson regression model in order to check the consistency of the association of each variable with disabling hearing loss, controlling by the others.

The data was analyzed using the Statistical Package for Social Sciences for Windows (SPSS) version 14.0 and STATA Data Analysis and Statistical Software version 9.2.

## RESULTS

Among the 349 households interviewed, 238 had between one and three dwellers and 1,050 subjects were analyzed. To avoid estimate biases in the analysis of the results, we weighed the data by age and gender, in such a way as to obtain the necessary equivalence between sample and population^13^.

The sample distribution by age range and gender was compared to that of the Brazilian population (2010)^14^ and it is depicted on Table 1.

**Table 1:** Ratio of the Brazilian population (2010) and the sample, sorted by age and gender.

Age range	Men		Women	
	Brazil	Sample	Brazil	Sample
0 to 9	15.7%	14.8%	14.5%	13.0%
10 to 19	18.5%	16.6%	17.3%	14.8%
20 to 29	18.3%	18.4%	17.7%	17.0%
30 to 39	15.5%	15.0%	15.6%	14.8%
40 to 49	12.9%	14.0%	13.2%	14.8%
50 to 59	9.4%	10.6%	9.9%	11.8%
60 to 69	5.6%	5.8%	6.3%	7.1%
70 to 79	3.0%	3.2%	3.6%	4.3%
80 and over	1.2%	1.4%	1.9%	2.4%

The hearing level of the populational sample, assessed by means of the TOAEs or audiometry and distributed by age and degree of hearing loss is depicted on Table 2. All the children between 0-3 years did not have DHL.

**Table 2:** Number of individuals (n) classified in function of the hearing level of the best ear and age. Juiz de Fora, MG, Brazil 2011.

				Disabling hearing loss		
Age range (years)	n	No loss (0-25 dB)	Mild loss (26-30 or 40 dB)	Moderate (31 or 41-60 dB)	Severe (61-80 dB)	Profound (> 80 dB)
0-3	52	52	0	0	0	0
4-9	94	91	2	1		
10-19	173	167	1	3	1	1
20-29	153	148	2		2	1
30-39	113	105	4	3		1
40-49	151	140	9	2		
50-59	135	103	25	5	2	
60-69	89	45	33	10		1
70-79	54	18	24	11	1	
≥ 80	36	3	8	18	7	
Overall total	1.050	872	108	53	13	4

Concerning background noise, we found a general mean value of 58.95 dBHL (CI = 58.55 dB to 59.35 dB); 58.94 dBHL in the DHL group and 58.95 dBHL in the group without DHL (*p* = 0.995).

**Figure 1:**
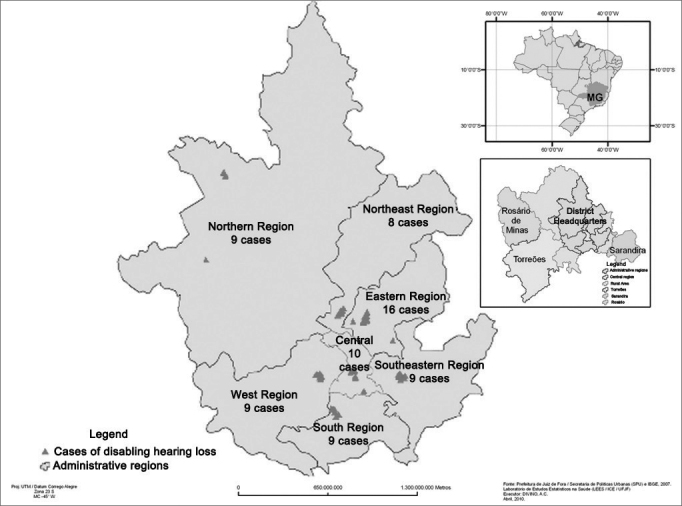
Distribution of the individuals with DHL among the randomly chosen sectors in the corresponding administrative regions of Juiz de Fora, MG, Brazil, 2011 (n = 70).

The administrative area of Juiz de Fora is divided into seven regions. We found nine cases of DHL in the northern region; eight in the northeast; 16 in the eastern; 10 in the central; nine in the west; nine in the south and nine in the southeastern region, making up a total of 70 cases as per depicted on Figure 1. Table 3 shows the results of the associations of the numerous factors with DHL, for the bivariate analysis of both regression models.

**Table 3:** Bivariate analysis and Poisson regression models 1 and 2, regarding the association of the DHL-related variables in the studied population of the city of Juiz de Fora - MG, Brazil, 2011.

DHL-related variables	Chi-squared (x^2^)				Model 1*				Model 2#			
	PR	CI 95%		*P*	PR	CI 95%		*P*	PR	CI 95%		*P*
Age range	8.621	5.263	14.286	< 0.001	-	-	-	-	7.42	4.33	12.73	< 0.001
Race	1.502	0.859	2.625	0.149	-	-	-	-	-	-	-	-
Gender	1.154	0.690	1.930	0.585	-	-	-	-	-	-	-	-
Educational level	2.298	1.285	4.108	0.004	2.12	1.26	3.55	0.004	1.91	1.13	3.25	0.016
Tinnitus	3.021	1.724	5.291	< 0.001	2.41	1.43	4.18	0.002	2.45	1.39	4.32	0.002
High blood pressure	4.115	2.481	6.803	< 0.001	3.46	2.10	5.71	< 0.001	-	-	-	-
DM	1.500	0.475	4.464	0.513	-	-	-	-	-	-	-	-
Smoking	1.900	1.068	3.425	0.028	-	-	-	-	-	-	-	-

PR: Prevalence ratio. LI: Confidence interval lower threshold. LS: Confidence interval upper threshold. CI: Confidence interval and *p*: statistical significance measure. HAS: High blood pressure. DHL: Disabling Hearing loss.

* Poisson Regression Model included with the variables which were significant considering the chi-square test without the Age variable.

# Poisson Regression Model including all significant variables and the Age variable.

DHL prevalence was estimated to be 5.2% (95% CI = 3.1-7.3). DHL was classified as moderate in 3.9% (95% CI = 0.001-0.134), severe in 0.9% (95% CI = 0.001-0.107) and profound in 0.4% (95% CI = 0.001-0.095).

## DISCUSSION

When we compare the data from this sample with that from the Brazilian population. we find relevant similarities. The male and female distribution in the age ranges portray the Brazilian population in general^14^.

Background noise was assessed in all the homes according to the ABNT technical standard^15^ (Brazilian Association of Technical Standards) 10.152 and it did not impact the results. The noise found where the audiometric tests were carried out did not show statistically significant difference between the mean level of noise found in the home of the people with and without DHL.

In Brazil, an emerging country with HDI (Human Development Index) of 0.699 (PNDU. 2010)^16^, the concern with studies about DHL prevalence is a relatively new thing. This study was the first to investigate the prevalence of DHL in southeastern Brazil, done with through a WHO protocol. The Brazilian southeastern is the most economically developed region of the country, encompassing 10.86% of the national territory, with 44% of the Brazilian population, with the three largest states population-wise^17^.

DHL prevalence in Juiz de Fora was estimated in 5.2% (95% CI = 3.1-7.3). This estimate is lower than the data found in one study carried out in Canoas - in southern Brazil, in which this prevalence was estimated to be 6.8%. This reduction in prevalence is probably due to governmental programs such as vaccination campaigns, early detection of hearing loss efforts carried out in maternities - such as the Universal Neonate Hearing Screening (UNHS), free hearing aid distribution program, and other measures^7^.

It is worth stressing that there may be a correlation between DHL prevalences in the world vis-à -vis the HDI of the country; for instance, it would be expected to find lower DHL prevalence in countries with high HDI. This can be seen in Denmark, for instance, where the HDI is high (0.866), with 0.2% of DHL prevalence^18^. Notwithstanding, in countries with medium HDI, such as Thailand (HDI: 0.654) and Sri Lanka (HDI: 0.658) had DHL prevalences of 13.6% and 9.0%, respectively^19^. On the other hand, a poor African country, such as Serra Leoa, with a very low HDI (0.317) has a DHL prevalence estimated to be 1.15%, lower than that of developed countries, such as the USA - with a high HDI (0.956) which DHL prevalence was estimated in 1.2%^20^. This is in disagreement with information on African hearing health found in the literature^6^, ^21^.

Even in today's age we still lack accurate estimates concerning DHL prevalences based on objective criteria, especially in developing countries, because of the poor investments made in epidemiological research^6^.

When we compare the estimate prevalence of DHL in the present study (5.2%) with that of European countries, such as Denmark, with 0.2%^18^; United Kingdom with 3.9%^22^; Sweden with 3.3%^23^, we may conclude that DHL prevalence in southeastern Brazil can be considered high. Nonetheless, when compared to the DHL prevalence of India - 10%^24^; in Nicaragua - 18%^25^; and Mexico - 19%^26^; the one estimated here (5.2%) was much lower.

After we finished estimating the DHL prevalence in the city of Juiz de Fora, we georeferenced the cases. We found more cases in the eastern region, when compared to the other regions, though without statistical significance (*p* = 0.131) (Figure 1).

The bivariate analysis and the prevalence ratio estimate between the DHL and the many variables alone was carried out before entering the Poisson regression model. Results are depicted below (Table 3).

DHL prevalence in the population with more than 60 years was six times higher than that of the younger individuals. Mader^27^ defines aging HL as the combined effect of presbycusis, socioacusis and occupational noise. In agreement with Agrawal et al.^28^. who found an increase in HL prevalence with age in all the groups; and Souza et al.^12^, who reported a correlation between presbycusis and age increase, male gender, diabetes mellitus and family history of presbycusis. As far as age is concerned, in Australia they found a DHL prevalence of 13.4% in the age range between 55 and 99 years, and the DHL prevalence in this study with subjects older than 60 years was estimated in 23.4%. In the USA, in patients with more than 65 years, they estimated a DHL prevalence of 30%.

DHL prevalence in caucasian individuals was 50% higher when compared to non-caucasians. Bunch^29^ reported that presbycusis affected the population in the following order: white men > white women > black men > black women; and such findings have been reinforced by other authors^12^, ^30^.

Hearing loss was 15% higher among men, when compared to women - which is in agreement with the report from Bainbridge et al.^20^, who stressed the risk of HL when associated with male gender, low educational level, factory or military occupation, free times of exposure to noise and smoking. The greater prevalence among males is corroborated by other authors^28^, ^30^.

DHL among individuals with lower educational level (up to elementary education) was 130% greater when compared to individuals with higher educational levels. One study carried out in Norway corroborates such data. There, they found a DHL prevalence 60% higher among less qualified workers and manual laborers when compared to more skilled laborers^31^.

Among subjects with tinnitus, DHL was 202% greater than among the others. This result is similar to that from Martines et al.^32^, in which the authors suggest a DHL-induced auditory pathway reorganization in these individuals, which could be one of the main sources of tinnitus sensation. Deafferentation and neuroplasticity are mechanisms which also contribute to this process. Ahmad & Seidman^33^ also suggest that the damage responsible for the many factors with play a role in the onset and worsening of DHL tend to increase with age and are associated with a worsening in tinnitus.

Among individuals with high blood pressure, DHL was 311% higher when compared to those without it. Kannel^34^ reports a high incidence of stroke in hypertensive patients. Przewoźny et al.^35^ reported that high blood pressure, lacunar stroke and bilateral ischemia are important risk factors for hearing loss in stroke patients. These data are reinforced by Cruisckshanks et al.^36^ and contrary to the findings reported by Souza et al.^12^.

DHL prevalence among smokers was 90% higher when compared to non-smokers. As far as smoking is concerned, in the beginning we did not find statistical significance in the bivariate analysis **(*p*** = 0.22). Nonetheless, after adjusting the ratios in function of gender and age range, the association with DHL became significant (*p* = 0.02). This finding is reinforced by one study carried out in India, where they found differences in the TOAEs of smokers^37^ and confirmed by Cruisckshanks et al.^36^ who, in a study, showed that smokers had twice the likelihood of developing DHL than non-smokers. Notwithstanding, the association between smoking and dyslipidemias seems to worsen even more the DHL evolution, as well as a past of high blood pressure alone or in association with other risk factors - which may worsen the problem.

Stopping smoking, reducing noise exposure and an efficient treatment for DM and high blood pressure may delay HL onset^28^.

DHL was 50% higher among individuals with diabetes than in those with normal glucose levels, and such result is in agreement with those from Bainbridge et al.^20^, who found a higher HL prevalence among diabetic patients; suggesting that HL may be an unknown complication of diabetes.

In the first model we included the variables which were significant in the bivariate analysis (chi-square test). Table 3 depicts the significant variables in the multivariate model. Smoking lost its significance when included in Model 1, and the variables which kept their significance were: arterial hypertension, educational level and tinnitus. In Model 1 we did not include the age variable, because it is a confounding factor regarding the association of the other variables and DHL. Adding it to Model 2 aimed at checking how the association of the other variables to DHL would change. We noticed that the inclusion of the age variable substantially affected the association between the DHL and numerous factors previously seen.

Since some DHL-associated variables tested are strongly related to age, in elderly individuals these factors are more frequent, just like the hearing loss. It is possible that as they are introduced in the model together with the age factor, the significance of the association between these variables and DHL comes down significantly. In Model 2, besides the age factor, the other significant variables were tinnitus and low educational level. These individuals had a DHL prevalence of 145% and 91% higher than the others, respectively. The results from this model indicated that the DHL prevalence among people older than 60 years was approximately six times higher than the others (Table 2).

Distribution by age range and gender in the sample was compared to that of the Brazilian population (2010)^14^, in which they found similarities among them. Thus, we can assume that the results from this paper could be similar to those found in the Brazilian setting.

We need more studies about the prevalence of DHL, especially in developing countries. Basic healthcare and prevention are determining factors for the productive future and quality of life of potential bearers of hearing disorders. Epidemiological knowledge concerning local and regional needs, considering environmental, genetic and cultural issues helps optimize investments and the implementation of planned health surveillance actions.

## CONCLUSION

DHL prevalence for the Juiz de Fora population was estimated to be 5.2%. The relationship among the many socioeconomic variables (age, gender, race and family income), cultural (educational level), comorbidities (DM, high blood pressure, tinnitus and smoking) and DHL found in the present study depicted a higher DHL prevalence in those older than 60 years of age, with low educational level, and who had tinnitus. The other variables correlated with DHL did not show statistical significance. We did not find a geographically determined risk area for DHL in Juiz de Fora.
